# Developmental exposure to indoor flame retardants and hypothalamic molecular signatures: Sex-dependent reprogramming of lipid homeostasis

**DOI:** 10.3389/fendo.2022.997304

**Published:** 2022-09-30

**Authors:** Elena V. Kozlova, Maximillian E. Denys, Jonathan Benedum, Matthew C. Valdez, Dave Enriquez, Anthony E. Bishay, Bhuvaneswari D. Chinthirla, Edward Truong, Julia M. Krum, Nicholas V. DiPatrizio, Poonamjot Deol, Manuela Martins-Green, Margarita C. Curras-Collazo

**Affiliations:** ^1^ Department of Molecular, Cell & Systems Biology, University of California, Riverside, Riverside, CA, United States; ^2^ Neuroscience Graduate Program, University of California, Riverside, Riverside, CA, United States; ^3^ Biomedical Sciences, School of Medicine, University of California, Riverside, Riverside, CA, United States

**Keywords:** metabolic syndrome & type II diabetes, polybrominated diphenyl ethers (PBDEs), maternal, leptin - adiponectin, fatty liver, endocrine-disrupting chemical (EDC), hypothalamus, dyslipidemia (DLP)

## Abstract

Polybrominated diphenyl ethers (PBDEs) are a class of flame-retardant organohalogen pollutants that act as endocrine/neuroendocrine disrupting chemicals (EDCs). In humans, exposure to brominated flame retardants (BFR) or other environmentally persistent organic pollutants (POPs) such as polychlorinated biphenyls (PCBs) and novel organophosphate flame retardants has been associated with increasing trends of diabetes and metabolic disease. However, the effects of PBDEs on metabolic processes and their associated sex-dependent features are poorly understood. The metabolic-disrupting effects of perinatal exposure to industrial penta-PBDE mixture, DE-71, on male and female progeny of C57BL/6N mouse dams were examined in adulthood. Dams were exposed to environmentally relevant doses of PBDEs daily for 10 weeks (*p.o.*): 0.1 (L-DE-71) and 0.4 mg/kg/d (H-DE-71) and offspring parameters were compared to corn oil vehicle controls (VEH/CON). The following lipid metabolism indices were measured: plasma cholesterol, triglycerides, adiponectin, leptin, and liver lipids. L-DE-71 female offspring were particularly affected, showing hypercholesterolemia, elevated liver lipids and fasting plasma leptin as compared to same-sex VEH/CON, while L- and H-DE-71 male F1 only showed reduced plasma adiponectin. Using the quantitative Folch method, we found that mean liver lipid content was significantly elevated in L-DE-71 female offspring compared to controls. Oil Red O staining revealed fatty liver in female offspring and dams. General measures of adiposity, body weight, white and brown adipose tissue (BAT), and lean and fat mass were weighed or measured using EchoMRI. DE-71 did not produce abnormal adiposity, but decreased BAT depots in L-DE-71 females and males relative to same-sex VEH/CON. To begin to address potential central mechanisms of deregulated lipid metabolism, we used RT-qPCR to quantitate expression of hypothalamic genes in energy-regulating circuits that control lipid homeostasis. Both doses of DE-71 sex-dependently downregulated hypothalamic expression of *Lepr*, *Stat3*, *Mc4r*, *Agrp*, *Gshr* in female offspring while H-DE-71 downregulated *Npy* in exposed females relative to VEH/CON. In contrast, exposed male offspring displayed upregulated *Stat3* and *Mc4r*. Intestinal barrier integrity was measured using FITC-dextran since it can lead to systemic inflammation that leads to liver damage and metabolic disease, but was not affected by DE-71 exposure. These findings indicate that maternal transfer of PBDEs disproportionately endangers female offspring to lipid metabolic reprogramming that may exaggerate risk for adult metabolic disease.

## Introduction

Metabolic syndrome (MetS) is a cluster of several conditions defined by the International Diabetes Federation to include: obesity, hypertension, dyslipidemia and hyperglycemia (1). MetS is increasing in an epidemic-like fashion, affecting 20-25% of the adult population worldwide, placing a significant economic burden on individuals and society (2, 3). While the prevalence of MetS and type II diabetes (T2D) has been attributed to the sedentary lifestyles and unhealthy diets of industrialized societies in the 20th century, other factors may be contributing to the etiology and pathophysiology of this epidemic, including exposure to endocrine disrupting chemicals (EDCs). EDC exposure during highly vulnerable life stages such as early development may increase the risk of disease later in life and in subsequent generations (4).

One class of compounds with endocrine disrupting properties are polybrominated diphenyl ethers (PBDEs), which have been increasingly used as additives in household products, furniture, clothing, toys and electronics since the 1970’s. After their adverse effects became known, three commercial mixtures of PBDEs, the penta-, octa-, and deca-BDEs were banned by the European Union and all PBDEs were voluntarily withdrawn by manufacturers in the US by 2013. In 2021, an unprecedented action by the US Environmental Protection Agency (US-EPA), formally banned deca-BDEs, Despite these bans, PBDEs continue to be released from existing products and environmental PBDE levels may be increasing due to their persistence, inadvertent recycling and e-waste (5, 6), PBDEs are ubiquitous and still being detected in various tissue samples (7–9). Therefore, PBDEs remain a public health concern and the long-term health consequences of exposure warrant further study.

Of the 75+ brominated flame retardants (BFRs) in circulation, PBDEs are of particular concern for diabetes and MetS due to their disproportionate developmental efficacy and lipid accumulating properties (10–15). Developmental PBDE exposure has been shown to produce marked and long-lasting effects on diabetic markers but these studies were mostly performed in male rodents. Recently, we reported that DE-71, a penta mixture of PBDEs, maternally transferred to offspring at 0.1 mg/kg/d, produces a diabetogenic phenotype characterized by several clinically used parameters, in females (16).

The potential effects of BFRs on adiposity and weight gain in developmentally exposed children are just becoming known. A screen of POPs and BFRs in human serum samples showed a significant association with T2D and MetS with two (PBB-153 and PBDE-153) out of six BFRs tested (17), suggesting that brominated POPs, stored in adipose tissue, may be involved in the pathogenesis of diabetes and MetS. Sexually dimorphic findings related to maternal serum BDE-153 levels during pregnancy were associated with high body mass index (BMI) in 7-year-old boys, but lower scores in 7-year-old girls (18). An inverse association was also found between the child’s own PBDE-153 concentration and BMI at 7 years of age, with no difference by sex.

The discovery that PBDEs activate and/or interact with signaling components involved in obesogenic pathways has spurred numerous studies on the effects of PBDEs on adipogenesis or lipogenesis outcomes in rodent and *in vitro* models (19–23). Evidence linking early-life exposure to PBDEs (BDE-47), with other MetS parameters is supported by a limited number of studies reporting exaggerated liver triglycerides and lipids and altered transcriptome related to lipid and glucose metabolism in male rats (10). Using a double hit HFD and BDE-47 model Wang (2018) found impaired lipid metabolism in male mice. However, there is limited information available about the effects of developmental PBDE exposure on lipid balance of females (24). Therefore, the purpose of this study was to compare the susceptibility of male and female offspring to the effect of developmental PBDE exposure. We measured peripheral parameters controlling lipid metabolism such as adipokines and plasma and liver markers of lipid metabolism.

Homeostatic regulation of feeding behaviors and energy balance is predominantly controlled by nutrient-sensing and endocrine-responsive signaling pathways in the hypothalamus (25, 26). The hypothalamus consists of a group of nuclei that communicate with each other to coordinate the organism’s energy state *via* metabolic hormones originating in white adipose tissue (WAT), stomach and pancreas (leptin, insulin, ghrelin) and nutrients (glucose, fatty acids) (27). For example, the arcuate nucleus, located near the blood brain barrier, detects internal and peripheral energy signals such as glucose, insulin, leptin and ghrelin *via* specific receptors for these molecules (28). Circuits in the arcuate work with those in the lateral hypothalamus and hypothalamic ventromedial nucleus and autonomic brainstem to control feeding, autonomic regulation of metabolism and energy expenditure (28, 29). Two hypothalamic regulatory pathways perform opposing functions: one consisting of neurons co-expressing Agouti-related protein (AgRP) and Neuropeptide Y (NPY), which stimulate food intake, and the other set of neurons co-expressing proopiomelanocortin (POMC) and cocaine- and amphetamine-regulated transcript (CART), which suppress food intake with presumed additional effects on energy expenditure and adipocyte lipolysis (30). Reduced feeding and increased energy expenditure is stimulated by peripheral leptin, a satiety hormone, secreted by WAT *via* activation of POMC/CART and inhibition of NPY/AgRP neurons. Together with the lipogenic hormone ghrelin, leptin targets the central melanocortin system, including its receptor, Mc4r, to modulate adipocyte and liver metabolism (31). The hypothalamus interacts with the autonomic nervous system which helps maintain energy balance in response to such signals, by regulating lipid metabolism in WAT, thermogenesis in brown adipose tissue (BAT), liver glucose production, pancreatic insulin secretion, and glucose uptake in skeletal muscle. Because hypothalamic control of energy homeostasis is regulated by these hormones signaling through receptors found here, these may be vulnerable to disruption by EDCs. PBDEs may interact with hypothalamic energy-sensing indicators/receptors and/or peripheral metabolic tissues and disrupt their complex balance leading to pathological states such as T2D and MetS. In the current study, we investigated the potential effect of perinatal exposure to DE-71 on plasma lipids and adipokines, hepatic lipid storage, and adiposity. We also studied putative central mechanisms involving hypothalamic markers of energy homeostasis as well as potential changes in gut barrier integrity. Our experimental design included males and female offspring as well as their DE-71 exposed mothers. We have attempted to integrate our findings in to an adverse outcomes pathway.

## Materials and methods

### Animal care and maintenance

C57Bl/6 N mice were generated using breeders obtained from Charles River Labs (West Sacramento, CA, USA) and were maintained in accordance with the guidelines in the National Institutes of Health *Guide for the Care and Use of Laboratory Animals* (32). Mice were group-housed 3-4 per cage in standard polycarbonate plastic cages with corn cob bedding unless otherwise noted. Food pellets (Laboratory Rodent Diet 5001; LabDiet, Quakertown, PA, USA) and municipal tap water were provided *ad libitum* except during the experimental period. Temperature was maintained at 21.1–22.8°C and relative humidity fluctuated between 20–70% under a 12/12 h photoperiod (lights on from 07:00–19:00 h). All experiments were approved by the IACUC on animal care and use at the University of California, Riverside.

### PBDE exposure

Offspring were exposed to the penta-brominated PBDE commercial mixture, DE-71, daily *via* maternal transfer using a 10-week dosing regimen as described previously (16). In brief, mice were randomly assigned to one of the three exposure groups: corn oil vehicle control (VEH/CON), L-DE-71 or H-DE-71. DE-71 dosing solutions were prepared in corn oil vehicle to yield two doses: 0.1 mg/kg/d (L-DE-71) and 0.4 mg/kg/d (H-DE-71) using 2 mL of stock solution/kg body weight. Dams were fed oral treats, (Kellogg’s Corn Flakes) infused with dosing solution daily, except on PND 0 and 1. Consumption was visually confirmed and offspring co-housed with dams were never observed to ingest cornflakes. The DE-71 doses were selected to contain the same molar concentrations of BDE-47 used in other mouse studies (24, 33). This exposure regimen involving maternal transfer to offspring yields accumulation of BDE congeners in offspring at ppm concentrations in liver (.2-1.0) and brain (.07-.3) (16, 34). In toddlers, ∑PBDE values in plasma have been reported to range from .1-.5 ppm. (35, 36). The published EPA reference dose for penta mixture of PBDEs (DE-71), is 2x10^-3^ mg/kg/day or 2 ppb (37).

Dams were dosed daily for a duration of 12 (70-80 d) weeks, which encompassed 3 weeks of gestation and 3 weeks of lactation until pup weaning at postnatal day (PND) 21. Female and male offspring were exposed during the *in utero* (GD 0-18) and lactational periods (PND 0-21) for approximately 39 d. Body composition measurements were performed at PND 54-80 in offspring and PND 160-180 in dams. Intestinal permeability assay was performed at PND 140-190 in offspring and PND 110-300 in dams. At necropsy, fat depots were excised and weights recorded and blood and organ tissue were collected for further analysis: liver lipids and histology, blood plasma hormones *via* ELISAs, liver enzymatic activity and hypothalamic gene markers *via* RT-qPCR.

### Measurement of body composition

Whole body composition was determined in live, unanesthetized mice by use of quantitative magnetic resonance (QMR) system, which relies on nuclear magnetic resonance (NMR) technology (EchoMRI; Echo Medical Systems, Houston, TX, USA) (38). Using various pulse sequences, the QMR system provides estimates of fat and lean tissue mass, which were expressed at % body weight. Duplicate QMR scans with accumulation times of 2 min were performed by placing previously-weighed mice into a well-ventilated plastic cylinder (1.5 mm thick, 4.7 cm inner diameter), with a cylindrical plastic insert added to limit movement. While in the tube, animals were briefly subjected to a low-intensity (0.05 Tesla) electromagnetic field.

### Tissue harvest

On the day of sacrifice, mice were anesthetized using brief CO_2_ inhalation followed by isoflurane inhalation, and blood was collected *via* cardiac puncture within 2-3 min. Plasma was isolated from blood, stored at -80°C and later used for immunoassays. At necropsy the following fat tissue depots were excised and weighed: interscapular BAT, mesenteric and inguinal WAT. Sample weights were normalized to body weight.

### Leptin and adiponectin enzyme immunoassays

Animals were subjected to a 12 h ON fast on wood chip bedding (SaniChip, P.J. Murphy Forest Products Corp., Montville, NJ, USA). Plasma collected from tail blood was analyzed for leptin and adiponectin using commercially available kits according to manufacturer’s instructions. Plasma leptin was measured using a commercial ELISA kit (ALPCO, Cat. #22-LEPMS-E01, Salem, NH, USA) having a standard range of 0.025-1.6 ng/mL. Therefore, we considered the accurate sensitivity to be the lowest standard, 0.025 ng/mL. Plasma Adiponectin was measured using a kit from Raybiotech (Cat. #ELM-Adiponectin, Norcross, GA, USA). The kit had an analytical sensitivity of 49 pg/mL in a standard range of 49-12,000 pg/mL. The colorimetric reaction products were read as optical density at 450 nm on a microplate reader (Molecular Devices, San Jose, CA, USA). Plasma leptin and adiponectin concentrations were determined by interpolating absorbance values using a linear standard curve.

### Plasma lipid analysis

Blood collected *via* cardiac puncture at sacrifice (*ad libitum fed state*) was assayed for plasma total cholesterol (high- and low-density lipoprotein) and triglyceride levels using commercially available fluorometric (total cholesterol) and colorimetric (triglyceride) kits (Cayman Cat. #10007640 & 10010303, Ann Arbor, MI, USA) according to the manufacturer’s instructions. The cholesterol assay had a sensitivity of 1.1 uM in a standard range of 2-20 uM. The triglyceride kit had a sensitivity of 0.5 mg/dL in a standard range of 3.125-200 mg/dL. Plasma cholesterol and triglyceride concentrations were determined by interpolating absorbance values using a linear standard curve.

### Liver lipid analysis

A modified Folch method was used to quantify total hepatic lipid content. Flash-frozen tissue samples (75-100 mg) were weighed, homogenized in 1 mL of methanol and vortexed followed by the addition of 2 mL of chloroform, 1 ml of water, and centrifugation at 3000 rpm for 15 min at 4°C in pre-weighed glass vials. The lower organic phase was transferred to a clean vial and the upper phase subjected to another chloroform extraction. The pooled lower phases were dried under N_2_ gas for 30 min at 37°C and the final lyophilized mass weighed and normalized to starting wet weight.

### Oil-Red-O (ORO) staining

At harvest, tissues were rinsed in phosphate-buffered saline (PBS) and immersion-fixed in 4% paraformaldehyde for 24 h at 4°C. After fixation, tissues were cryoprotected by two successive incubations in 20 and 30% sucrose (in 1X phosphate buffer) and flash-frozen in Tissue-Tek embedding medium over dry ice. Frozen liver tissue was cryosectioned at 7-12 μm. Liver sections were rehydrated with 60% isopropanol, stained for 15 min in freshly made 0.5% Oil-Red-O (ORO) stain (Sigma, St. Louis, MO, USA; Cat# 09755) in 3:2 solution of isopropanol:ddH2O). The slides were rinsed with 60% isopropanol, counter-stained to identify hepatocyte nuclei with modified Harris hematoxylin (Ricca Chemical, Cat# 3530-16, Arlington, TX, USA) for 90 sec, submerged in tap water for 3 min, followed by rinsing for 30 sec in distilled water and mounted with glycerin jelly consisting of gelatin, glycerol and phenol (IHC world). ORO lysochrome stains neutral lipids- triglycerides and cholesterol ethers (but not biological membranes) represented as red droplets (objects) in spaces between hepatocytes (39, 40). Images were captured at 40X using bright field optics (Nikon TMS, Tokyo, Japan) and digital camera (Spot Insight U3.2).

### Computer assisted densitometry of Oil-Red-O staining

For each animal, images were acquired of 3-5 microscope fields from each liver with a 40X objective using brightfield optics. All images to be compared were acquired using identical microscope settings and were used to quantify the ORO positive signal using open source software [QuPath v0.2.3 (41)]. For each sample, 3-5 subfields were selected, each designated as a region of interest (ROI). The total ORO positive area was summed for each subfield and normalized to ROI area. The normalized ORO positive area values were averaged and reported as mean ± sem.

### Intestinal permeability assay

Mice were fasted overnight (14-16 h) on wood chip bedding. Mice were gavaged with 60 mg/100 g b.w. of a 100 mg/mL of dextran conjugated to fluorescein isothiocyanate (FITC; 60 mg/100 g b.w.; 3-5 kDa; Sigma-Aldrich, Cat#: FD4) solution prepared in sterile 1X PBS. Twenty percent more of the calculated individual dose was given to each mouse to compensate for the volume the gavage needle retains. After 4 h, mice were euthanized under isoflurane anesthesia and blood (300–400 μL) collected *via* cardiac puncture into sterile tubes on ice. Serum was collected after cold-centrifugation at 2000 g for 15 min and diluted 1:5 in double-distilled water. Plasma FITC was measured in duplicate or triplicate spectrophotometrically (GloMax Promega, USA) in 96-well plates (excitation: 490/510–570 nm, 525/580–640 nm). FITC-dextran concentration of samples was determined from a standard curve generated using a diluted fluorophore stock gavage solution in the plasma matrix from fasted untreated mice. Three technical replicates of each sample were run.

### Administration of dextran sodium sulfate (DSS)

Dextran sodium sulfate (DSS), a water-soluble, negatively charged sulfated polysaccharide containing ~19% sulfur with a highly variable molecular weight ranging from (MW = 36,000–50,000 Da) is often used to induce breakdown of gut integrity leading a form of mouse colitis that mimics the clinical and histological features of irritable bowel disease (IBD) including ulcerative colitis (42). Colitis was established by the daily oral administration of 2-3% DSS (colitis grade, MP Biosciences) *via* drinking water, leading to inflammation and leaky gut in the mid-distal colon within 5-8 days of treatment. The solution was prepared fresh every 4 d. DSS mice were used as positive controls for gut permeability measurements in FITC-dextran experiments described above after 6-7 d post-treatment.

### Brain extraction and RNA isolation

At sacrifice, using isoflurane anesthesia and cervical dislocation, whole hypothalami were rapidly dissected and snap-frozen in 2-methylbutane over dry ice. The hypothalamus was hemissected and one-half portions immediately homogenized in TRIzol Reagent (Ambion by Life Technologies, Carlsbad, CA, USA) using a hand-held homogenizer. Total RNA was prepared *via* a guanidinium thiocyanate–phenol–chloroform extraction followed by manufacturer’s instructions, including DNAase1 treatment (Monarch Total RNA Miniprep Kit, #T2010, New England Biolabs, Ipswich, MA, USA and QIAGEN RNeasyMini, #74104, Dusseldorf, Germany). Purity and quantity of RNA were assessed by determining the optical density (OD) photometrically using 260/280 and 260/230 nm ratios (NanoDrop ND-2000, Thermo-Fisher Scientific Inc., Waltham, MA, USA).

### Quantitative polymerase chain reaction

RT-qPCR was used to quantitate mRNA transcripts for genes: *Npy, Lepr, Gshr, Stat3, Mc4r, Agrp*. Custom- or pre-designed DNA oligonucleotide PCR primers were obtained from Integrated DNA Technologies (Coralville, IA, USA). Primers were designed to meet several criteria using NCBI Primer Blast and then optimized by testing against complementary DNA generated using RT-PCR and gel electrophoresis. Only primers that gave single-band amplicons in the presence of reverse transcriptase (RT) and that matched the base length of the predicted target were selected. In addition, primers selected yielded 91.5 to 107.8% efficiency by RT-qPCR validation (**Table 1**). A temperature gradient was used to capture ideal annealing temperatures for custom designed primer pairs. For all primers, intercalating dye (SYBR green) chemistry was used. The primer concentration ranged from 200-500 nM.

**Table 1:** RT-qPCR primer sequences and efficiencies and PCR products.

Target gene	Gene symbol	GenBank accession number	Primer sequence Forward 5’-3’Reverse 5’-3’	Exon target (Fwd/ Rv)	E (%)	Tm^o^C (Fwd/ Rv)	Product length (bp)	Anneal temp (°C)
Agouti-related neuropeptide^a^	*Agrp*	NM_007427	AAGACAACTGCAGACCGAGC GCTAGGTGCGACTACAGAGG	4/5	95.9	57.7/57.0	244	60
Beta actin^b^	*Actb*	NM_007393	GATTACTGCTCTGGCTCCTAG GACTCATCGTACTCCTGCTTG	5/6	99.6	55.0/54.4	147	60
Growth hormone secretagogue receptor	*Ghsr*	NM_177330	CAGGCTCGAAAGACTTGGAAA ACCAGAACCACAAACAGACAG	2/3	91.5	58.5/58.4	114	60
Leptin receptor	*Lepr*	NM_010704	GCCGGTGTGAGTTTTCAGTC GTGCCATTGTTTGGCTGTCC	5/6	93.8	56.4/57.4	139	56
Melanocortin-4 receptor^c^	*Mc4r*	NM_016977	TGAACTTCTGAGAGGCTGCG TTCTCGGTTGACCAGTCTGC	N/A	99.3	57.2/57.2	175	60
Neuropeptide Y	*Npy*	NM_023456	TCACAGAGGCACCCAGA CACACGAGCAGAGATAGAGC	1/2	107.8	55.7/55.0	121	60
Signal transducer and activator of transcription 3	*Stat3*	NM_011486	ACCACGAAAGTCAGGTTGCT TGTGTTCGTGCCCAGAATGT	10/11-13	99.7	56.9/57	138	56

Fwd, forward; Rv, reverse; E, primer efficiency; Tm, melting temperature; bp; base pair. Primer sequences were obtained from: ^a^ (44); ^b^ (34); ^c^ (45). N/A, near upstream in-frame codon.

RT-qPCR was performed on RNA samples (40 ng), run in triplicate, on a CFX Connect or CFX96 thermocycler (Bio-Rad, Irvine, CA, USA) with the Luna Universal one-step qPCR Master Mix (E3005; New England Biolabs, Ispwich, MA, USA). Amplification reactions for genes of interest were performed in 50 cycles of the following cycling protocol: reverse transcription 55°C/10 min; initial denaturation 95°C/1 min; per cycle: 95°C/10 s denaturation, 60°C or 55°C/30 s extension; 65-95°C in 0.5°C, 5s increments melt curve analysis. In each experiment, no-template controls (NTCs) were run without mRNA to rule out extraneous nucleic acid contamination and primer dimer formation. Negative RT controls, which contained the complete set of RNA synthesis reaction components without the addition of the enzyme reverse transcriptase were used to rule out presence of genomic DNA (gDNA). Relative gene expression was measured relative to the reference gene, *ActB*, and differential gene expression was determined compared to the null group (VEH/CON) using the Pfaffl method (43). We have previously determined that DE-71 does not interfere with the expression of *ActB*.

### Statistical analysis

Normal distribution and homogeneity of variance of the data were tested. All statistical analyses were conducted using GraphPad Prism (GraphPad Software version 9.4.1, San Diego, CA, USA). All values are expressed as mean ± s.e.m. One-Way ANOVA was used to compare the effects of exposure. Brown-Forsythe ANOVA was used when variances were significantly different. Where the F ratio was significant, *post-hoc* comparisons were completed using Tukey’s, Dunnet’s or Sidak’s *post-hoc* test. Differences were considered statistically significant at *p*<0.05.

## Results

### Chronic DE-71 exposure has minimal effects on body composition

In order to ascertain the effects of DE-71 on body composition, Echo MRI was used to measure total fat and lean mass and body weight in exposed and control mice. The results are summarized in **Table 2**. There were no differences in adult male or female offspring nor dams. However, there was an apparent decrease in relative lean mass in H-DE-71 female offspring relative to VEH/CON (One-way ANOVA F_(2,41)_=2.72, *p*=0.08, Dunnett’s *post-hoc* VEH/CON vs H-DE-71, *p*=0.06). No changes were observed in this cohort, although we found a decrease in body weight in L-DE-71 female offspring previously (16).

**Table 2:** Chronic DE-71 exposure has minimal effects on body composition.

	VEH/CON	L-DE-71	H-DE-71
**Female offspring**			
n	20	16	8
Body Weight	18.4 ± 0.27	18.3 ± 0.22	17.3 ± 0.66
Fat Mass Absolute Relative	2.02 ± 0.06 11.0 ± 0.27	2.08 ± 0.06 11.4 ± 0.36	1.83 ± 0.15 10.5 ± 0.80
Lean Mass Absolute Relative	15.6 ± 0.21 84.9 ± 0.33	15.3 ± 0.21 84.1 ± 0.32	14.4 ± 0.56 83.4 ± 0.86^i^
**Male offspring**			
n	26	26	16
Body Weight	22.2 ± 0.20	23.0 ± 0.26	22.0 ± 0.29
Fat Mass Absolute Relative	2.40 ± 0.10 10.8 ± 0.42	2.36 ± 0.09 10.2 ± 0.30	2.23 ± 0.09 10.1 ± 0.39
Lean Mass Absolute Relative	19.0 ± 0.15 86.0 ± 0.54	19.7 ± 0.21 85.9 ± 0.32	19.1 ± 0.28 86.9 ± 0.52
**Dams**			
n	7	6	5
Body Weight	24.8 ± 1.1	24.4 ± 0.83	24.1 ± 0.71
Fat Mass Absolute Relative	2.78 ± 0.27 11.1 ± 0.64	2.93 ± 0.37 11.8 ± 1.1	2.79 ± 0.14 11.6 ± 0.35
Lean Mass Absolute Relative	21.2 ± 0.81 85.3 ± 0.75	20.5 ± 0.62 84.0 ± 0.93	20.7 ± 0.64 86.0 ± 0.89

Body and absolute fat mass weights are in grams; fat mass-to-body-weight ratios (relative weights) are given as g fat weight/g body weight. Values are reported as mean ± s.e.m.

i Indicates an apparent difference relative to VEH/CON (p < 0.06) by Dunnet’s post hoc test following One-way ANOVA (F_(2,41)_ = 2.724, p = 0.08).

### Chronic exposure to DE-71 reduces thermogenic BAT in female and male offspring

**Figure 1:**
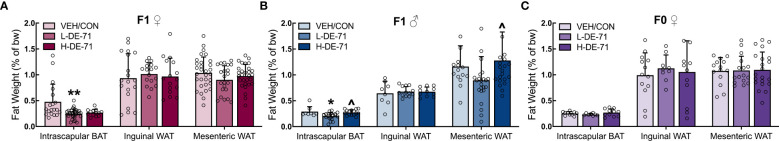
Brown adipose tissue (BAT) in DE-71-exposed male and female offspring. **(A–C)** Normalized weight of fat depots (WAT and BAT) were compared across treatment groups: 0.1 mg/kg/d (L-DE-71), 0.4 mg/kg/d (H-DE-71) and oil vehicle controls (VEH/CON) of female **(A)**, male **(B)**, and dams **(C)**. *p<0.05, **p<0.01 compared to VEH/CON; ^*p*<0.05 compared to L-DE-71. Sample size (n/group): **(A)** female offspring 16-31/group; **(B)** male offspring 8-24/group; **(C)** dams 8-21/group. Bars and error bars represent mean ± s.e.m. BAT, brown adipose tissue. WAT, white adipose tissue [*Permission obtained for reuse of left panel in **Figure 2A** and left panel in**2C**, Kozlova et al, 2020, Sci. Rep. 10:18102]*.

**Figure 2:**
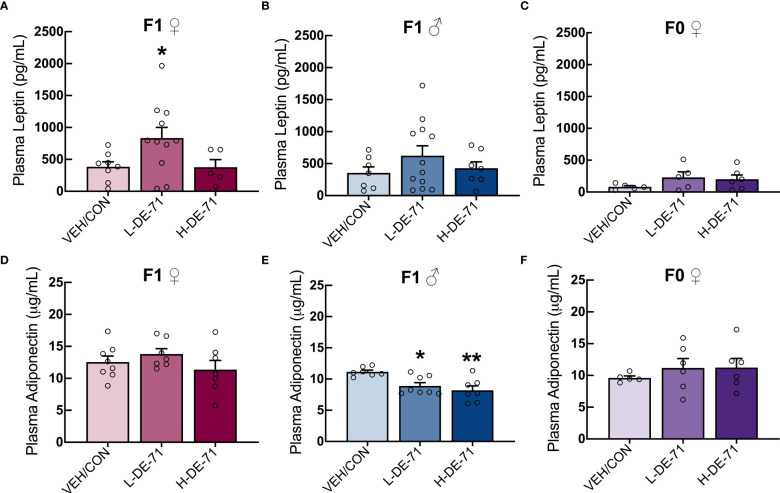
Plasma levels of leptin and adiponectin in DE-71-exposed male and female offspring. Female and male offspring were fasted for 12 h and plasma collected and analyzed using commercial ELISA kits. Samples were also processed for dams in *ad libitum* fed state. **(A–C)** Mean plasma leptin levels in DE-71-exposed and VEH/CON groups. **(D–F)** Mean plasma adiponectin levels DE-71-exposed and VEH/CON groups. Asterisk(s) indicate compared to VEH/CON, *p<0.05, **p<0.01. Sample size (n/group): **(A)** female offspring, 6-11, **(B)** male offspring, 7-12, **(C)** dams, 5-6, **(D)** female offspring, 7-8, **(E)** male offspring, 7-8, **(F)** dams, 5-6.

Obesity is defined largely by body mass index (BMI); however, physiological evidence indicates that body fat distribution, irrespective of BMI, most strongly predicts the risk of obesity-associated disease (46). Therefore, we examined the fat stores in interscapular BAT, inguinal WAT and mesenteric WAT. **Figure 2** shows decreased BAT in L-DE-71 female and L-DE-71 male offspring (**Figures 1A, B**) (Females: Brown-Forsythe ANOVA F_(2,21.2)_=7.448, *p*=0.0035, Dunnett’s *post-hoc* VEH/CON vs L-DE-71, *p*=0.02; Males: One-Way ANOVA F_(2,41)_=6.82, *p*=0.0028, Tukey’s *post-hoc* VEH/CON vs L-DE-71, *p*=0.01, L-DE-71 vs H-DE-71, *p*=0.01. No group differences were observed in dams (**Figure 1C**).

### DE-71 exposure disrupts plasma leptin and adiponectin in a sexually dimorphic manner

Leptin, as a single factor, is associated with increased cardiometabolic risk and predicts MetS (47). Adiponectin, secreted from WAT, decreases liver lipids and deficiency of adiponectin is associated with insulin resistance, T2D and cardiac mortality (48). Therefore, we measured plasma levels of adiponectin and leptin in a fasted state using commercial immunoassay kits. Our results show that, relative to VEH/CON, L-DE-71 female offspring display elevated plasma leptin: Brown-Forsythe ANOVA: F_(2,18.8)_=5.8, *p*<0.05; Dunnet’s *post-hoc* VEH/CON vs L-DE-71 *p*=0.04 (**Figure 2A**). No group differences were observed in male offspring or dams (**Figures 2B, C**). For adiponectin L- and H-DE-71 male offspring displayed significantly lower levels as compared to VEH/CON: One way ANOVA: F_(2,19)_=8.224, *p*<.0.01; Tukey’s *post-hoc* VEH/CON vs L-DE-71 *p*=0.05, vs H-DE-71 *p*<.01 (**Figure 2E**). No group differences were observed in female offspring or dams (**Figures 2D, F**).

### L-DE-71 exposure elevates plasma total cholesterol in a sexually dimorphic manner without affecting triglycerides

**Figure 3:**
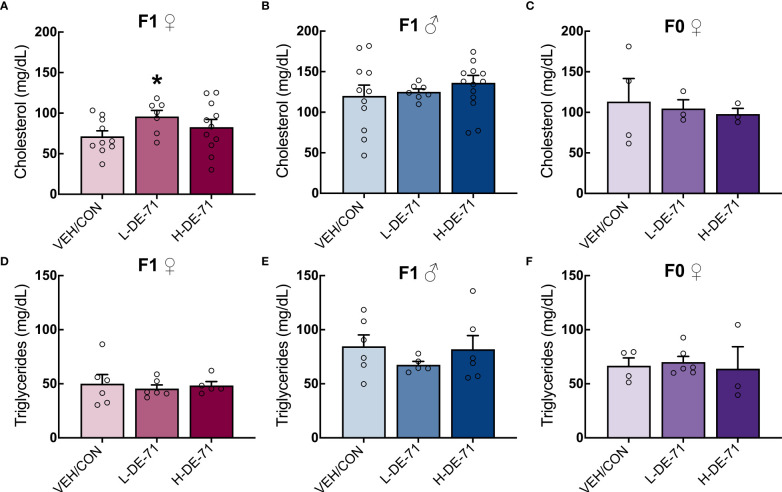
Effect of perinatal L-DE-71 exposure on plasma total cholesterol and triglycerides in DE-71-exposed male and female offspring. **(A–C)** Plasma total cholesterol in female **(A)** and male **(B)** offspring and dams **(C)**. **(D–F)** Plasma triglyceride levels in female **(A)** and male **(B)** offspring and dams **(C)**. *p<0.05 compared to VEH/CON; Bars and error bars represent mean ± s.e.m. Sample size (n/group): **(A–C)** females, 7-11; males, 7-14; dams, 3-4. **(D–F)** females, 5-6; males, 5-6; dams, 3-6.

In order to evaluate lipid metabolism, we examined plasma levels of total cholesterol and triglyceride since high total of LDL cholesterol, low HDL cholesterol and high triglycerides are indicators of dyslipidemia and risk factors for cardiovascular disease and other comorbidities (49). A significant elevation in plasma total cholesterol was observed in female L-DE-71 offspring compared to VEH/CON (One-Way ANOVA F_(2,41)_=6.818, *p*=0.0028, Tukey’s *post-hoc* VEH/CON vs L-DE-71, *p*=0.0112) (**Figure 3A**). No group differences were observed in male offspring nor dams (**Figures 3B, C**). Triglyceride levels were similar across DE-71 exposure groups in male and female offspring and dams (**Figures 3D–F**) as previously reported for BDE-47 exposed male offspring (10). These results reveal a sexually dimorphic effect of PBDEs on cholesterol levels, with exposed female offspring showing a unique susceptibility to L-DE-71.

### L-DE-71 exposure produces fatty liver in female offspring

**Figure 4:**
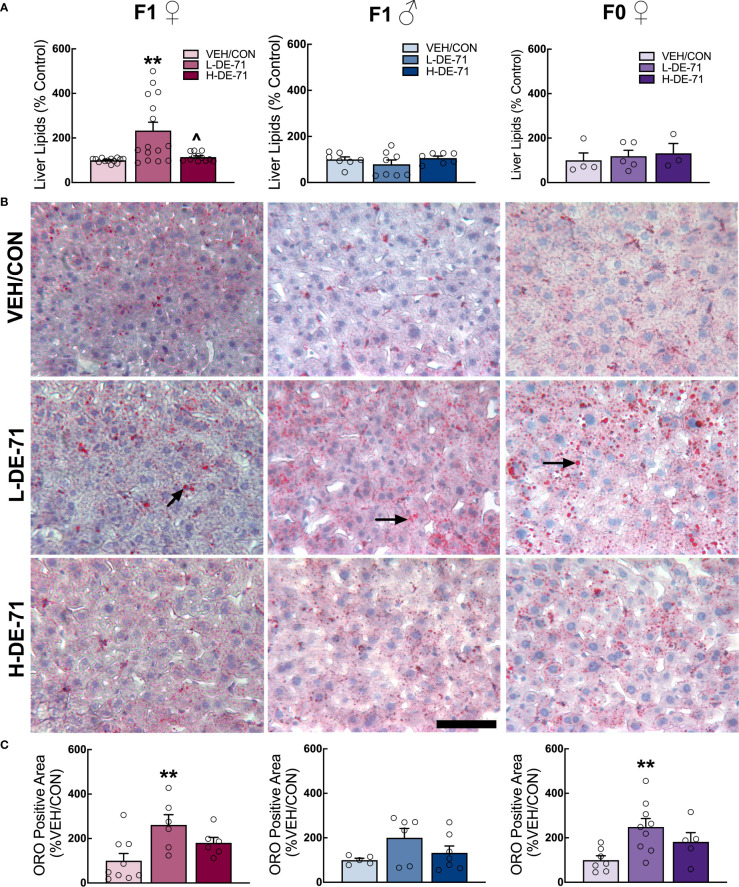
Sex-dependent effects of perinatal exposure to L-DE-71 on liver lipids in male and female offspring. **(A)** Total hepatic liver lipids expressed as percent VEH/CON. **(B)** Representative micrographs of liver samples processed for Oil Red O staining show lipid globules (black arrows) on hematoxylin stained hepatocytes. **(C)** Densitometric quantification in ORO-stained livers. **compared to VEH/CON*, p*<0.01. Bars and error bars represent mean ± s.e.m. Sample size (n/group): **(A)** female offspring, 12-15; male offspring, 5-8; dams, 5-9. **(C)** female offspring, 6-9; male offspring, 5-6; dams, 4-8. Scale bar, 100 microns.

The excessive accumulation of lipids in the liver may be a risk factor for the development of liver steatohepatitis, the most prevalent chronic liver disease with a widespread prevalence of 25% in the adult population (50). Liver steatohepatitis is also a risk factor for markedly greater mortality and morbidities like MetS (51), T2D, obesity, dyslipidemia, hypertension and cardiovascular disease (51). We, therefore, examined the effects of DE-71 on liver lipid content by processing snap frozen liver tissue using the Folch method. Results in **Figure 4** show mean normalized lipid levels are significantly elevated in L-DE-71 exposed female offspring as compared to VEH/CON (**Figure 4A**). Mean raw lipid concentrations were 204, 249, 231 mg lipid/g tissue for VEH/CON, L-DE-71 and H-DE-71, respectively. A Brown-Forsythe ANOVA revealed a statistically significant effect of exposure (F_(2,14.7)_ =11.08), *p*=0.001. Dunnett’s T3 *post-hoc* showed differences between L-DE-71 and VEH/CON females (*p*=0.01) as well as between L-DE71 and H-DE-71 females (*p*=0.02). No differences were observed in males or dams (**Figure 4A**).

In another subset of mice, we performed ORO histochemistry on cryosectioned liver tissue sections. Microscope analysis revealed bright red circular-stained structures, indicating fat deposits (**Figure 4B**). Densitometric quantification indicated that L-DE-71 produced additional fat deposits in exposed female but not male offspring as compared to their respective VEH/CON group (**Figure 4C**). Although studied at different exposure conditions, previous reports indicate increased ORO staining in developmentally exposed male rodent offspring when exposed to higher doses of DE-71 (50 mg/kg, Wistar Han rats) (52); or to a specific BDE congener, BDE-47 (1 mg/kg, CD-1 mice) (10). L-DE-71 dams also showed more numerous lipid globules as compared to VEH/CON (**Figure 4C**). In agreement with adult susceptibility to altered hepatic fat storage after direct PBDE exposure (BDE-47, 1 mg/kg), greater ORO staining was reported for adult male rats (11). With regard to Folch measured lipids and ORO staining, previous reports on females and quantitative analysis of PBDE-induced liver lipids are lacking.

### Normal gut integrity after developmental or adult exposure of DE-71

**Figure 5:**
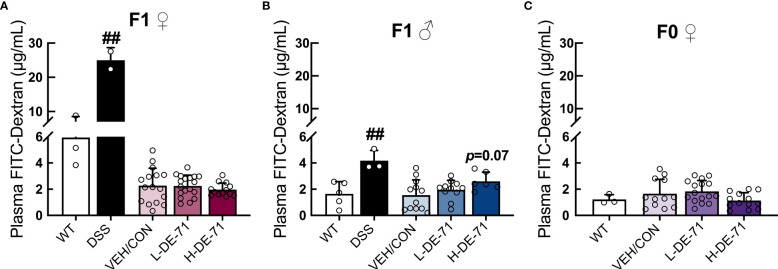
Gut barrier permeability in DE-71 exposed dams and offspring. Mice were fasted 14 h before gavage with 60mg/kg of 4 kDa FITC-dextran and serum samples collected 4 h later were read for fluorescence intensity at 490 nm using a Promega GloMax spectrometer. **(A–C)** Plasma FITC levels in exposed and VEH/CON groups. Bars and error bars represent mean ± s.e.m. Exposure had no main effect as indicated by One-way ANOVA. DSS treatment was used as a positive control; ^##^significantly different compared to VEH/CON, *p*<0.01, Student’s t-test. Sample size (n/group): **(A)** female offspring 2-16. **(B)** male offspring: 3-12. **(C)** dams 3-16.

Intestinal permeability was also examined, since intestinal-derived bacterial products entering the bloodstream may cause inflammation and promote liver disease that may contribute to obesity and diabetes (53). To explore whether BDEs in DE-71 produce metabolic effects in parallel with gut inflammation, we evaluated gut barrier integrity by subjecting mice to an oral dextran-FITC permeability test. Plasma FITC levels were analyzed 4 h later using fluorimetry. Our results, shown in **Figures 5A–C**, indicate normal gut permeability across all experimental groups (Male: Brown-Forsythe ANOVA, F_(2,23.2)_=2.97, *p*=0.07; Female offspring: F_(2,29.5)_=0.48, *p*=0.62; Dam: One-way ANOVA, F_(2,38)_=2.5, *p*=0.09). H-DE-71 male offspring showed an apparent increase in intestinal leakage (*p*=0.07). We included an experimental group exposed to dextran sodium sulfate (DSS), a chemical colitogen with anticoagulant properties, to induce disease, that was used as a positive control and expectedly found a 154 and 356% greater FITC leakage into plasma than VEH/CON in male and female offspring, respectively (unpaired t-test, *p*<0.01).

### Sexually dimorphic effects of DE-71 on expression of hypothalamic energy balance genes

**Figure 6:**
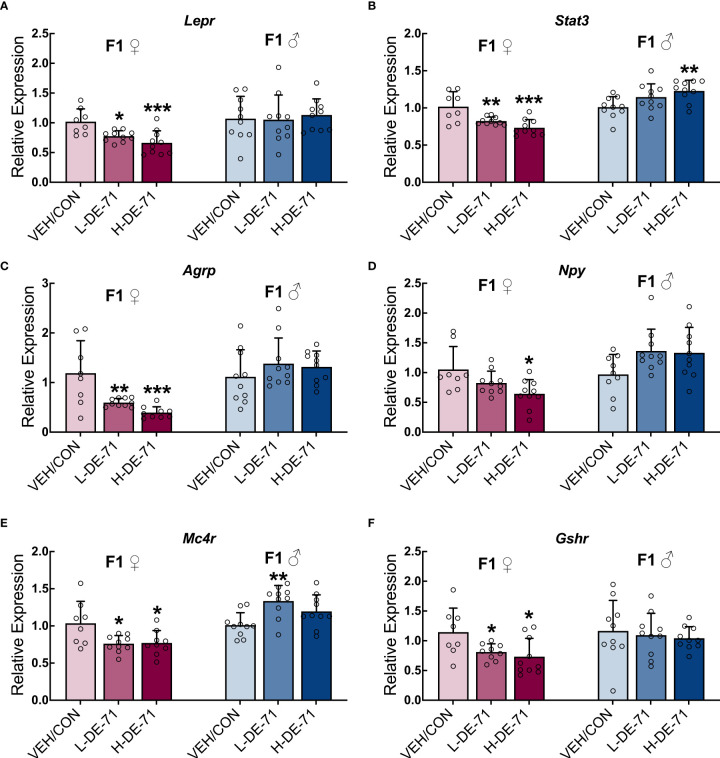
Sex-dependent alterations of hypothalamic energy balance genes in DE-71-exposed F1 offspring. Relative gene expression in hypothalamic samples of adult male and female offspring from dams orally dosed with vehicle or DE-71 was measured using RT-qPCR and mean values normalized to *Actb* and expressed as fold-change relative to the mean of respective VEH/CON samples. **(A)** Leptin receptor, *Lepr* **(B)** Signal transducer and activator of transcription 3, *Stat3* **(C)** Agouti-related peptide, *Agrp* **(D)** Neuropeptide Y, *Npy* **(E)** Melanocortin-4 receptor, *Mc4r* **(F)** ​​​​Growth hormone secretagogue receptor, *Gshr*. Data are represented mean ± s.e.m. Sample size (n/group): Females (8-10) and males (9-10). Asterisks indicates significant difference compared to VEH/CON, *p<0.05, **p<0.01, ***p<0.001.

The regulation of energy balance and body composition is a complex process that involves interaction between the central and peripheral systems. We explored the effects of developmental exposure on hypothalamic gene markers for energy-sensing and signaling important for regulation of energy balance. **Figures 6A–F** shows the mean fold change in hypothalamic RNA samples of exposed offspring relative to VEH/CON. Overall, DE-71-exposed female offspring showed abnormal *downregulation of all* genes tested. Specifically, exposed females showed reduced *Lepr, Stat3, Agrp, Npy, Mc4r* and *Gshr.* One-way ANOVA values ranged between *p*<0.05 to *p*<0.001. The greatest reduction occurred in *Agrp* in L-DE-71 (49.8 *±* 4.3%) and H-DE-71 (66.9 *±* 10.0%) in female offspring (*p*<0.001, n=8-10/group). In contrast, males showed changes in *only two* genes (*Stat3* and *Mc4r*) and these were both *upregulated* (*p*<0.05 in H-DE-71 and *p*<0.01 in L-DE-71, respectively, n=9-10/group). This is consistent with the more pronounced lipid metabolic reprogramming characterized in L-DE-71 exposed female offspring. However, abnormal gene expression occurred in both L-DE-71 and H-DE-71 mice, indicating a lack of dependency on DE-71 dose.

## Discussion

The present study provides the first evidence of an environmentally relevant PBDE mixture on lipid metabolism and potential associated central mechanisms (34). Specifically, lipid parameters in exposed female offspring were compared to those obtained from their male counterparts and their mothers. Female offspring of dams fed L-DE-71 (0.1 mg/kg/d) are susceptible to develop hypercholesterolemia, fatty liver, elevated plasma leptin and altered central markers of energy homeostatis relative to same-sex vehicle controls. Notably, female mice exposed to the greater dose of DE-71 (0.4 mg/kg/day) were not symptomatic. This is in agreement with the non-monotonic dose response reported for other EDCs (54). In contrast, female adult dams lacked this phenotype, suggesting that developing females are more vulnerable to the endocrine- and metabolic-disrupting effects of PBDEs. Taken together, our findings indicate that central mechanisms involving hypothalamic markers of energy homeostasis as well as hepatic lipid storage, may, in combination, pose a unique risk to EDC-exposed female offspring for metabolic disorders.

### Plasma cholesterol

Perinatal exposure to L-DE-71 produced elevated plasma total cholesterol in female but not male offspring, relative to their respective vehicle controls (**Figure 3**), in spite of the fact that males display greater basal levels of cholesterol than female mice (55). Abnormal plasma cholesterol or triglycerides are one criteria of MetS, along with abdominal a diposity (56), elevated glycemia/diabetes and high blood pressure (47). While we did not detect changes in mesenteric fat (**Figure 1**), we have previously reported diabetogenic (increased fasting glycemia and glucose intolerance, insulin insensitivity) and exaggerated pressor responses in adult offspring of DE-71 exposed dams (16, 57). Hypercholesterolemia may follow from fatty liver phenotypic reprogramming (58) produced by PBDEs in DE-71 as speculated in the AOP. Recently, Tartu (2017), used metabolome and lipidome approaches to establish that PBDEs (along with PCBs, chlordanes and perfluoroalkyl substances) were significantly related to cholesterol homeostasis and other biomarkers involved in lipid accumulation, FA metabolism, and insulin utilization in Norwegian polar bears (59). In addition, altered adipocyte function may also contribute (23). For example, penta-BDE mixture promotes stimulated lipolysis and reduced insulin-stimulated glucose oxidation in adipocytes *in vitro* (60). Further study is needed to determine the source of elevated cholesterol and the initiating events triggered by PBDEs.

### Exaggerated hepatic lipid accumulation

Our previous mass spectroscopy results indicated persistent accumulation of BDE-28/33 and -153 in adult livers of DE-71 exposed female offspring, suggesting that livers are a target of BFRs such as PBDEs (16). The hypercholesterolemia observed in L-DE-71 females led us to quantify lipid content in the livers of all mice using the Folch method. Elevated hepatic lipid content was only displayed by L-DE-71 female offspring (**Figure 4**). This group also showed elevated ORO staining, which represents a partial set of lipids. Other studies in male mice have shown that perinatal/postnatal exposure to BDE-47 produces excess ORO staining and altered transcriptomics in liver pathways related to lipid and carbohydrate metabolism, insulin signaling and T2D (10, 61). Notably, upregulated cluster of differentiation (*Cd36) membrane protein* may indicate that PBDEs alter fatty acid uptake since *Cd36* has been associated with non-alcoholic fatty liver disease (NAFLD) in humans. In combination, our findings emphasize the risk for fatty liver in humans with high body burdens of PBDEs. NAFLD is the most common liver disease in world, affecting adults at epidemic proportions (25%), with risk factors ranging from age and sex to MetS and insulin resistance (62). Genetics alone cannot explain the large increase in the prevalence of NAFLD during the past 2 decades and the increase that is projected for the next decades. Developmental exposure to EDCs such as PBDEs may contribute added risk; a recently identified form of NAFLD is toxicant-associated fatty liver disease (TAFLD) in occupationally exposed workers (63). Unlike BDE-47, our findings show that DE-71 produces minor effects on liver lipids of developmentally exposed males compared to females. In our hands, exposed females but not males also show hallmarks of T2D such as elevated fasting glycemia, glucose intolerance, insulin resistance and reduced plasma insulin (16). Indeed, fatty liver and T2D share insulin resistance as their chief pathogenic determinant (64).

The abnormal hepatic lipid accumulation in L-DE-71 female phenotype occurred in the absence of excess fat depots, although lean mass showed an apparent decrease (**Table 2**). At first, this may appear inconsistent with fatty liver disease despite being the most common risk factor for NAFLD. In fact, up to 20% of Americans with normal BMI have NAFLD. Similarly, null associations were noted between concurrent PBDEs and BMI among adolescents from a cohort in the Netherlands (65) and between prenatal PBDEs and BMI in children of the Center for the Health Assessment of Mothers and Children of Salinas (CHAMACOS) study (15). Other epidemiological studies have reported inverse relationships with postnatal PBDE congeners −47, −100, −153, and −154 and adiposity in girls aged 6–8 years (66).

### Plasma leptin and adiponectin

Our immunoassay results demonstrate elevated fasting plasma leptin in L-DE-71 female offspring (**Figure 2**). Our findings of augmented circulating leptin, and fatty liver without increased adiposity are in agreement with previous studies. Leptin levels are positively associated with severity of NAFLD (47, 67), that cannot be ascribed to a corresponding elevation in WAT (**Figure 1**). The importance of our finding is further emphasized since elevated fasting leptin, as a single factor, is associated with increased cardiometabolic risk and predicts MetS. Leptin is also an important regulator of blood glucose levels (68). In contrast, a longitudinal study on a small sample of children in the Netherlands failed to show any correlation between leptin and toxicants (PBDEs, PCBs or dioxins) (65). Adiponectin was also studied because it is inversely linked with insulin resistance, lipid accumulation, inflammation and NAFLD and increasing plasma adiponectin is being tested as a new therapy for NAFLD (69). PBDE-exposed male offspring showed reduced plasma adiponectin but their liver lipids and plasma cholesterol were normal (**Figure 2**). In humans, a positive association has been found between ΣPBDE and fasting levels of adiponectin in older persons (70). More studies are needed to determine the endocrine disrupting effects of PBDEs and plasma adipokines.

### Central control of lipid metabolism

The role of the hypothalamus is also physiologically relevant to the abnormal lipid metabolic phenotype (excess hepatic lipid storage and hypercholesterolemia). We found a widespread effect of early-life exposure to PBDEs on energy-regulating gene markers in the hypothalamus (**Figure 6**). Notably, female offspring exposed to L-DE-71 display reduced *Lepr*, and its signaling partners *Stat3* and *Mc4r.* Together with chronically elevated fasting levels of leptin (**Figure 2**), the central changes may represent a form of leptin resistance that has not been previously reported EDC action of PBDEs. Downregulated *Lepr, Stat3* and *Mc4r* (see below) may alter effectiveness of peripheral leptin’s anorexigenic and lipid balancing homeostatic actions (lipolysis, decrease WAT mass and lipid accumulation). PBDE-induced elevation in circulating leptin levels may impair cholesterol removal in the diabetogenic phenotype of L-DE-71 females (16) as can happen to non-exposed animals under conditions of high blood glucose (71). Therefore, the aberrant lipid metabolic profile of L-DE-71 in female offspring (**Figure 4**) may be caused, in part, by abnormal leptin actions.

Leptin actions are, in part, mediated *via* inhibition of NPY/AgRP neurons (30, 72). Notably, neuropeptide gene expression of *Agrp* was also downregulated in L-DE-71 (and *Npy* in H-DE-71) female offspring. Female *Agrp* expression is also susceptible (opposite change) to perinatal organophosphate flame retardants (OPFR) exposure when combined with a high fat diet (73). In contrast to the results obtained on females, DE-71 exposed males showed *upregulated Stat3* and *Mc4r* without altered *Npy, Agrp*, *Ghsr*, or *Lepr.* For reference, others have reported *downregulated* hypothalamic NPY and POMC systems in male C57BJ/6 mice by adult exposure to other EDCs such as bisphenol A, diethylstilbestrol and tributyltin (74).

L-DE-71 female offspring also displayed downregulated expression of growth hormone secretagogue receptor (GHSR) (**Figure 6**) to which peripheral ghrelin binds and produces orexigenic and lipogenic actions (25, 75, 76). Both ghrelin and leptin target the central melanocortin system, including its receptor, MC4R, to modulate adipocyte and liver metabolism (77). Therefore, downregulated central levels of *Mc4r*, seen in L-DE-71 female offspring, may play a role in deregulating hypothalamic-mediated metabolic homeostasis (**Figure 6**). The central melanocortin system also modulates hepatic lipid metabolism; animals with reduced MC4R expression or function show increased lipogenesis rate and triglyceride content in the liver (78, 79). Therefore, changes seen in *Mc4r* may participate in the exaggerated liver lipid content, and hypercholesterolemia produced in females by developmental exposure to L-DE-71 (**Figure 3**). Other EDCs such as OPFRs that have replaced PBDEs in home environment, disrupt energy homeostasis through toxic actions on the hypothalamic melanocortin circuitry in a sex-dependent manner (80).

Developmental abnormalities of hypothalamic neural circuits can lead to body weight and metabolic imbalance (81) from neuroinflammation, as reported after air pollutant exposure (82). Whether organohalogens that have access to hypothalamus (83) produce their actions *via* similar neuroinflammation is still unresolved. One study has shown that perinatal polychlorinated biphenyls (0.5 mg/kg) can produce persistent changes in glial fibrillary acidic protein, a measure of neuroinflammatory response in brainstem and cerebellum, but hypothalamus was not tested (84). It should be noted that PBDEs have prominent disruptive effects on the thyroid hormone system, which controls lipid metabolism and inflammatory processes, and responds to leptin receptor activation (85).

### Putative adverse outcome pathway for metabolic reprogramming by PBDEs

**Figure 7:**
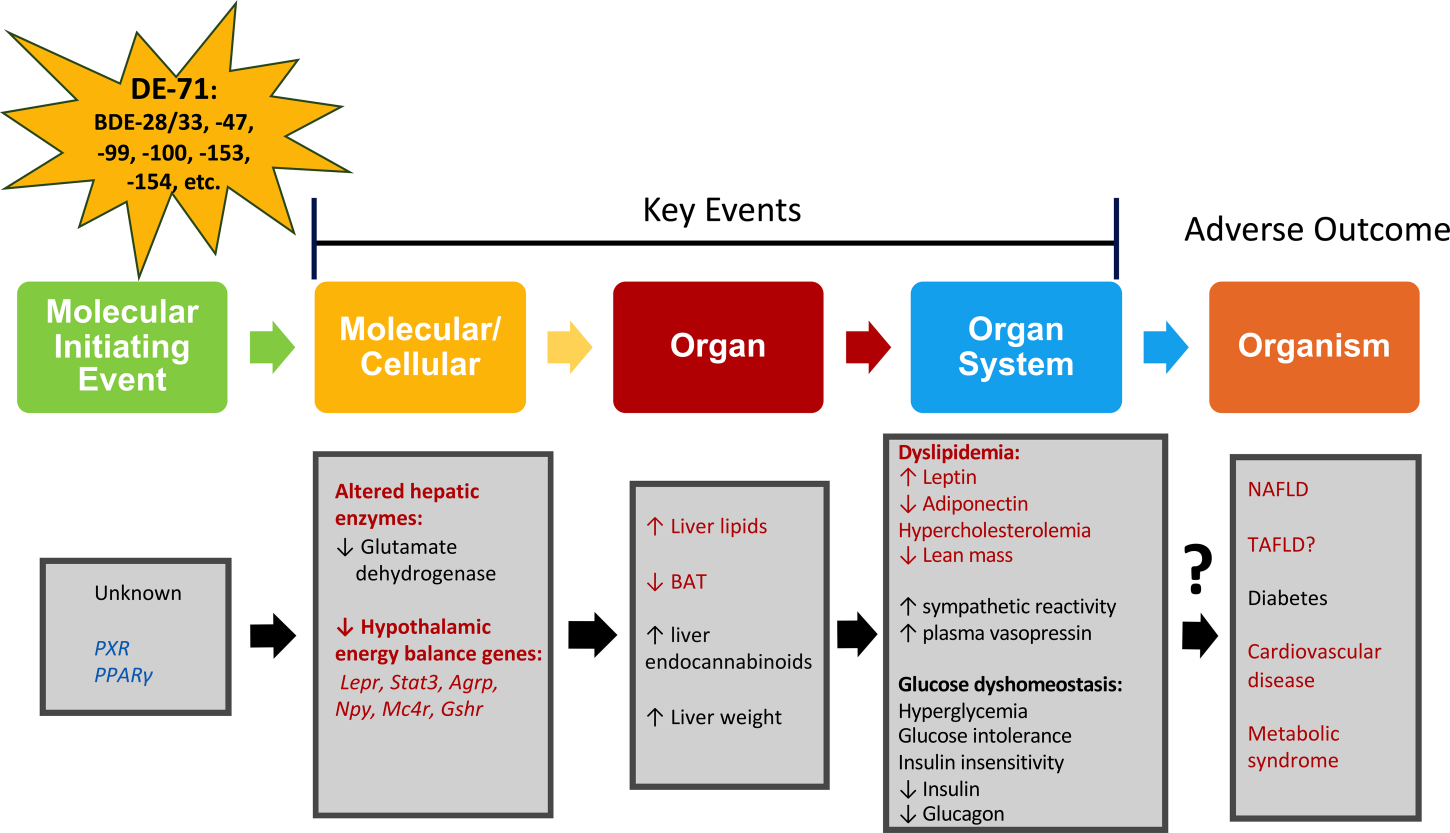
Adverse outcomes pathway (AOP) for metabolic alterations produced by DE-71. An AOP network portrays the plausible pathway from an initial perturbation by a penetrating toxicant called a molecular initiating event (MIE) to an adverse outcome, through different intermediate events called key events (KE) taking place at diverse levels of biological organization such as molecular, cellular, organ and organ system (93). Metrics evaluated are indicated in red (current study) and black (previously reported in (16, 57, 87). Four KEs leading to liver steatosis, diabetes, and metabolic syndrome are proposed: elevated hepatic lipid content, reduced expression of energy balance genes, increased plasma leptin, decreased plasma adiponectin. Blue text: MIE has not been determined but activation of pregnane X receptor (PXR) and peroxisome proliferator-activated receptors (PPARγ) has been suggested as part of drug-induced liver injury and steatosis (88, 89, 93). Hypothalamic energy balance genes: Leptin receptor, *Lepr*; Signal transducer and activator of transcription 3, *Stat3*; Agouti-related peptide, *Agrp;* Neuropeptide Y, *Npy;* Melanocortin-4 receptor, *Mc4r;* Growth hormone secretagogue receptor, *Gshr;* toxicant-associated fatty liver disease, TAFLD.

The lipid metabolic profile detected in female offspring perinatally exposed to L-DE-71 may indicate a propensity for MetS. In agreement with this broader assignment, we have previously shown that L-DE-71- exposed female offspring show a combination of diabetogenic features including fasting hyperglycemia, insulin insensitivity, glucose intolerance and altered glucoregulatory hormones. Female dams do not display altered hypercholesterolemia nor diabetogenic phenotype thus, adults are less susceptible to DE-71 exposure relative to offspring. Our previous findings also indicate elevated plasma vasopressin in L-DE-71 female offspring (34); elevated vasopressin (as measured by its stable surrogate marker copeptin) is positively associated with hepatic steatosis and MetS (86). The effects produced by developmental exposure to DE-71 have been incorporated into a possible adverse outcome pathway that describes key events leading to physiological and organismal outcomes such as MetS, T2D, fatty liver disease and cardiovascular disease (**Figure 7**). Exposure to DE-71, with primary constituents BDE-47 and BDE-99, triggers changes in liver gene expression with patterns matching that of metabolic syndrome (52, 90). BDE-47 concentrations have been associated with liver enzymes indicating hepatobiliary dysfunction (11, 14). While we did not investigate the possible participatory MIE(s) in **Figure 7**, a focus on nuclear receptors and aryl hydrocarbon signaling has been recommended on the basis of involvement in endocrine disruption by BFRs (91). There is evidence that BFRs like PBDEs activate the nuclear hormone receptors, Pregnane X receptor (PXR) and peroxisome proliferator-activated receptors (PPARγ), both transcriptional regulators of hepatic uptake/deposit of lipids (92–94). Indeed, PBDE-99 stimulates adipogenesis and increases PPARγ expression in other metabolic organs such as murine and human preadipocytes (20).

An aggravated inflammatory response in the liver may contribute to the elevated lipid content and dyslipidemic phenotype displayed by L-DE-71 exposed female offspring. In NAFLD livers, lipid elevation is associated with inflammation; however, the precise mechanism by which hepatic lipid accumulation and inflammation contribute to NAFLD remains poorly understood (95). We have previously shown elevated hepatic endocannabinoids in response to perinatal exposure to DE-71 in female offspring that may participate in lipid dyshomeostasis and inflammation (16). Notably, elevated N-arachidonoylethanolamine (AEA) can serve as a source of arachidonic acid but can also be metabolized by most eicosanoid biosynthetic enzymes, yielding bioactive lipids with pro-inflammatory actions (96). Although the release of proinflammatory arachidonic acid in response to PBDE exposure has not been studied in metabolic organs like liver or adipose tissue, DE-71, a mostly pentabromodiphenyl ether mixture, but not DE-79, a mostly octabromodiphenyl ether mixture, causes a dose-dependent increase in arachidonic acid release from rat neuronal cultures (97). Moreover, livers of DE-71 exposed females and dams show reduced glutamate dehydrogenase, which normally offsets fatty liver characteristics such as enhanced lipogenesis (and gluconeogenesis) (98). Mechanistic studies will be necessary to determine the cellular and biochemical targets of PBDEs that may predispose mammals to abnormal metabolic phenotypes (99).

### Sexually dimorphic effects of PBDEs

Our findings suggest that, in contrast to male offspring, the normal lipid balance of female offspring is uniquely susceptible to environmentally relevant doses and composition of PBDEs congeners. Given that the sex steroid environment and receptor distribution in metabolic tissue are distinct between males and females, it is conceivable that PBDEs’ mechanistic actions vary by sex (100). It is plausible that PBDEs have sexually dimorphic actions on biological processes since they are agonists of estrogen receptors and antagonists of androgen receptors (101, 102). Alternatively, it has been suggested that the findings of metabolic effect modification by sex of PBDEs may be partially explained by body fat and lean mass percentage rather than sexual dimorphism. For example, a case has been made that higher gains in fat mass (in females) relative to greater lean mass (in males) may yield disproportionate sex-dependent consequences of PBDEs (103). In our hands, DE-71 did not produce increased adiposity (fat mass, increased WAT or body weight) in any of the experimental groups studied (**Figure 1**; **Table 2**).

In this study, we did not detect elevated adiposity such as fat mass, increased WAT or body weight. In contrast, the specific effect of L-DE-71 on BAT (**Figure 1**) in female offspring is especially significant since the latter is now recognized as playing a significant role in controlling obesity and body glucose homeostasis (104). The significantly lower BAT measured in L-DE-71 female offspring relative to vehicle controls may secondarily result from significantly elevated catecholamine levels reported by our group (16). Indeed, increased catecholamine levels and catecholamine resistance resulted in reduced BAT (105), a state that clinical trials have been designed to oppose in order to alleviate metabolic diseases. The effects of PBDEs on BAT may be secondary to PBDE-triggered upregulation of sympathetic activity; the latter is supported by previous reports from our lab and others (57, 106). Elevated sympathetic activity could also contribute to changes in glycolipid metabolism measured here and in previous works (16). The adverse lipid metabolic implications of low developmental PBDE exposure seen in adulthood warrant further study into the underlying mechanisms that could be protected or suppressed by novel approaches. Since PBDEs impact metabolomic profiles related to lipid, carbohydrate and energy metabolism in association with gut dysbiosis (107), one such treatment could involve an anti-obesogenic probiotic therapy with lipolytic consequences (108).

## Conclusion

In conclusion, perinatal exposure to an environmentally relevant mixture/concentration of PBDEs during early life produces elevated fasting leptin, hypercholesterolemia and fatty liver in adult female offspring. In combination with the PBDE-induced diabetogenic phenotype characterized in our previous report (16), current findings suggest increased risk of MetS posed by BFRs like PBDEs. More research is critically needed to understand the molecular mechanisms and long-term adverse outcomes of early-life exposure to BFRs. Further concern is warranted by their sex-dependent effects, with developing females having greater susceptibility for metabolic disturbances, and the widespread environmental occurrence of legacy and emerging environmental flame retardants. The unique abnormal glucose and lipid metabolic phenotypes of exposed female offspring are not likely caused by gut inflammation as suggested by gut integrity experiments (**Figure 5**), but may result, in part, from abnormal expression of central gene markers in hypothalamic energy-regulating circuits. In particular, reduced hypothalamic *Lepr* and downstream STAT3 signaling with elevated fasting plasma levels of leptin may represent deregulation of the central control of lipid (and glucose) metabolism. Interestingly, L-DE-71-exposed females also show autism-relevant deficient social recognition and deregulated neuromolecular markers (34) indicating potential interactions between metabolic and behavioral disorders as speculated previously (109).

## Data availability statement

The original contributions presented in the study are included in the article/supplementary materials. Further inquiries can be directed to the corresponding author.

## Ethics statement

The animal study was reviewed and approved by The University of California, Riverside IACUC.

## Author contributions

Conceptualization, EK and MC-C; Methodology, EK, MD, MC-C, MV, ND, and PD; Validation, EK, MV, MD, and MC-C; Formal Analysis, AB, EK, MV, MD, and MC-C; Investigation, AB, BC, DE, EK, ET, JB, JK, MV, MD, MM-G, MC-C, and PD; Writing – Original Draft, EK, AB, MD, and MC-C; Writing – Reviewing and Editing, EK and MC-C; Visualization, EK and MC-C; Resources, MM-G, MC-C, and ND; Data Curation, AB, BC, EK, MD, and MC-C; Supervision, EK, MV, and MC-C; Project Administration, EK, MD, AB, and MC-C; Funding Acquisition, EK, MM-G, MC-C, and ND. All authors reviewed and approved the final manuscript.

## Funding

This work was supported by a University of California President’s Pre-Professoriate Fellowship and UCR CNAS HSI Undergraduate Research Program Fellowship to EK and UC Riverside Omnibus and Committee on Research (CoR) Grants, UCMEXUS and Academic Senate Committee on Research grants to MC-C.

## Acknowledgments

We thank Drs. Prue Talbot and David Carter for their help with microscopy. We also thank Karthik Basappa, Richard Martirosian, Dr. Clara Berdasco, Crystal Luna (Curras-Collazo Lab), Drs. Donovan Argueta and Pedro Perez (DiPatrizio lab), Anthony Castro, Yagnika Patel and Dr. Cristina Flores (Martins-Green lab), Dr. Anica Sayok, Rocio Alvarez and Dr. McCole (McCole lab), Dr. Hashini Batugedara and Dr. Meera Nair (Nair Lab) for their technical assistance. We are grateful to Great Lakes Corporation for the gift of DE-71 and to Drs. F. Sladek and I. Ethell for the gift of C57BL6 mice. We thank Dr. Djurdjica Coss for the use of plate reader (Promega Glo-Max Pro, Madison, WI, USA) and Dr. Sachiko Haga-Yamanaka for use of plate shaker. We thank D. Marvin (ALPCO) for adiponectin EIA kit.

## Conflict of interest

The authors declare that the research was conducted in the absence of any commercial or financial relationships that could be construed as a potential conflict of interest.

## Publisher’s note

All claims expressed in this article are solely those of the authors and do not necessarily represent those of their affiliated organizations, or those of the publisher, the editors and the reviewers. Any product that may be evaluated in this article, or claim that may be made by its manufacturer, is not guaranteed or endorsed by the publisher.

## Author disclaimer

JK is now a 2nd Lieutenant at the Uniformed Services University, Department of Defense. Her work was performed at the University of California, Riverside before becoming a military officer. However, we want to emphasize that the opinions and assertions expressed herein are those of the authors and do not necessarily reflect the official policy or position of the Uniformed Services University or the Department of Defense.

## Glossary

**Table:**

	
Agrp	agouti-related peptide
AOP	adverse outcome pathway
BAT	brown adipose tissue
BFR	brominated flame retardants
BMI	body mass index
DSS	dextran sodium sulfate
EDC	endocrine disrupting chemicals
FA	fatty acid
FITC	fluorescein isothiocyanate
FM 550	firemaster 550
gDNA	genomic DNA
Gshr	growth hormone secretagogue receptor
IBD	irritable bowel disease
IACUC	institutional animal care and use committee
Lepr	leptin receptor
Mc4r	melanocortin-4 receptor
MetS	metabolic syndrome
MIE	molecular initiating event
NAFLD	non-alcoholic fatty liver disease
NMR	nuclear magnetic resonance
Npy	neuropeptide Y
NTC	no-template control
ORO	Oil-red O
OD	optical density
PBB	polybrominated biphenyls
PBS	phosphate-buffered saline
PBDE	polybrominated diphenyl ether
PND	postnatal day
p.o.	per os
POP	persistent organic pollutants
PPARγ	peroxisome proliferator-activated receptors
PXR	pregnane x receptor
QMR	quantitative magnetic resonance
RT	reverse transcriptase
Stat3	signal transducer and activator of transcription 3
T2D	type II diabetes
TAFLD	toxic-associated fatty liver disease
WAT	white adipose tissue
